# Modeling and Optimizing the Effect of Light Color, Sodium Chloride and Glucose Concentration on Biomass Production and the Quality of *Arthrospira platensis* Using Response Surface Methodology (RSM)

**DOI:** 10.3390/life12030371

**Published:** 2022-03-03

**Authors:** Ahmad Nosratimovafagh, Abolghasem Esmaeili Fereidouni, Felix Krujatz

**Affiliations:** 1 Department of Fisheries Science, Faculty of Animal Sciences and Fisheries, Sari Agricultural Sciences and Natural Resources University (SANRU), Sari P.O. Box 578, Iran; a.nosrati@stu.sanru.ac.ir; 2 Institute of Natural Materials Technology, TU Dresden, Bergstraße 120, 01069 Dresden, Germany; Felix.Krujatz@tu-dresden.de; 3 biotopa gGmbH—Center for Applied Aquaculture & Bioeconomy, Bautzner Landstraße 45, 01454 Radeberg, Germany

**Keywords:** *Spirulina*, mixotrophic, salinity, light color, glucose, LED, response surface methodology, RSM

## Abstract

*Arthrospira platensis* (*Spirulina*) biomass is a valuable source of sustainable proteins, and the basis for new food and feed products. State-of-the-art production of *Spirulina* biomass in open pond systems only allows limited control of essential process parameters, such as light color, salinity control, or mixotrophic growth, due to the high risk of contaminations. Closed photobioreactors offer a highly controllable system to optimize all process parameters affecting *Spirulina* biomass production (quantity) and biomass composition (quality). However, a comprehensive analysis of the impact of light color, salinity effects, and mixotrophic growth modes of *Spirulina* biomass production has not been performed yet. In this study, Response Surface Methodology (RSM) was employed to develop statistical models, and define optimal mixotrophic process conditions yielding maximum quantitative biomass productivity and high-quality biomass composition related to cellular protein and phycocyanin content. The individual and interaction effects of 0, 5, 15, and 30 g/L of sodium chloride (S), and 0, 1.5, 2, and 2.5 g/L of glucose (G) in three costume-made LED panels (L) where the dominant color was white (W), red (R), and yellow (Y) were investigated in a full factorial design. *Spirulina* was cultivated in 200 mL cell culture flasks in different treatments, and data were collected at the end of the log growth phase. The lack-of-fit test showed that the cubic model was the most suitable to predict the biomass concentration and protein content, and the two-factor interaction (2FI) was preferred to predict the cellular phycocyanin content (*p* > 0.05). The reduced models were produced by excluding insignificant terms (*p* > 0.05). The experimental validation of the RSM optimization showed that the highest biomass concentration (1.09, 1.08, and 0.85 g/L), with improved phycocyanin content of 82.27, 59.47, 107 mg/g, and protein content of 46.18, 39.76, 53.16%, was obtained under the process parameter configuration WL4.28S2.5G, RL10.63S1.33G, and YL1.00S0.88G, respectively.

## 1. Introduction

There is an increasing demand for proteins, natural pigments, and lipids in the food, pharmaceutical, and aquaculture industries [1] that can be obtained in a sustainable way from microalgae biomass [2], as it does not interfere with animal feed or human food production. Therefore, these organisms are known as the flagship of the third generation of food resources [3].

*Arthrospira platensis* (*Spirulina*) is known as a superfood that contains valuable compounds, such as high-digestible proteins [4,5], vitamins [6], unsaturated fatty acids such as gamma-linolenic acid [7], and pigments, especially for the anti-cancer blue phycocyanin [8].

The biomass productivity and biochemical composition of *Spirulina* biomasses are highly dependent on the adjusted cultivation conditions, such as temperature [9], light quality (spectrum) [10,11,12] and quantity (intensity) [13,14], and the availability of nutrients [15]. *Spirulina* is commonly cultivated in large-scale open raceway ponds using natural sunlight. Despite the low biomass production costs using raceway ponds compared to closed photobioreactors, this way of production is associated with a lower biomass productivity, a high risk of contamination, and limited options for process control. Hence, attention has increasingly been paid to developing closed photobioreactors (PBRs) for boosting production [16], and more flexibility for exploiting the metabolites from *Spirulina* by optimizing the essential process conditions, such as light color, salinity, and availability of organic carbon sources. However, comprehensive studies investigating both the individual, as well as combinatorial, effects of these process conditions to control the quantity and quality of the product biomass have not yet been conducted. *Spirulina* offers a high metabolic flexibility, and can grow under photoautotrophic, heterotrophic, and mixotrophic conditions [12]. In heterotrophic cultures, a long lag phase, a low specific growth rate, and a decrease in the cellular phycocyanin content were reported [17], whereas under mixotrophic growth conditions, Marquez et al. showed that there was no significant lag phase [18], and the growth rate was equal to the sum of the photoautotrophic and heterotrophic growth [17]. Mixotrophic conditions can thus lead to an increase in quantitative biomass yield compared to photoautotrophic or heterotrophic conditions.

Salinity is one of the most important factors affecting growth and valuable cellular compounds in algae [19]. Due to the high tolerance of *Spirulina* towards various salinity conditions, the use of high salinity is considered as one way to control undesired contamination [20,21,22]. Most studies dealing with the salinity tolerance of *Spirulina* have been performed in photoautotrophic environments, with contradictory results [19,23,24,25,26]. In mixotrophic process conditions, high salinity was reported to have a negative effect on biomass production [27]. Due to a lack of knowledge on the interaction of trophic growth modes and salinity tolerance, further studies are necessary to specifically analyze how varying salinity in mixotrophic conditions affects the quantity and quality of *Spirulina* biomass production.

Light quality, i.e., the spectral composition of photosynthetic active radiation (PAR), is another important factor affecting biomass productivity and biomass composition. Due to the cell-specific pigmentation, phototrophic microorganisms, such as microalgae and cyanobacteria, are not able to absorb all spectral parts of PAR [28,29]. Among the various sources of artificial light, light-emitting diodes (LEDs) are characterized by a high level of efficiency, a long lifetime, low energy consumption, and the absence of toxic materials in their composition [30,31]. White, blue, red, green, yellow, and orange LEDs have been reported in previous studies to affect *Spirulina*, biomass productivity, and quality in terms of its chemical composition or pigment content [32,33,34,35,36,37]. For instance, red LED light improved the growth rate of *Spirulina* by 29–67%, whereas blue LED light increased the production of cellular fats, carbohydrates, and phycocyanin; however, the highest protein production was reported using green and white LED light [11,32,33,38,39,40]. Tian et al. [30] suggested that the presence of blue light is necessary to improve metabolic functions in photosynthesis. Red light is needed for the efficient operation of photosystems I (700 nm) and II (680 nm). Therefore, the concept of using different spectral parts of light was suggested to improve both the quantity and quality of algal biomass [30]. The most suitable spectral light composition for photoautotrophic *Spirulina* cultivation was suggested to be a combination of three LEDs emitting red, green, and blue light, with a ratio of 80:5:15, respectively [37]. However, Bachchhav et al. [34] reported that under mixotrophic conditions, yellow LEDs were more efficient than red LEDs, leading to the hypothesis that a combination of mixotrophic process conditions with balanced salinity, and LED light with dominant yellow spectral parts, can be beneficial for biomass production (quantity), as well as protein and phycocyanin content (quality).

Response Surface Methodology (RSM) is a mathematical and statistical technique to assess the effect of several independent variables on a respond of interest. Despite the basics of RSM dating back to the 1920s, this approach is widely-used in current research and process development in various disciplines, such as environmental biotechnology [41], bioprocess engineering, and wastewater treatment [42]. Breig and Luti 2021 [43] highlighted the power of RSM to optimize microbial production of primary and secondary metabolites. Karimifard and Moghaddam 2018 [44] presented RSM approaches in order to optimize the physicochemical wastewater treatment. Also, many recent studies successfully used the RSM approach to optimize *Spirulina* growth conditions for the enhancement of biomass productivity [45], air revitalization [46], developing screening photobioreactors [47], and phycocyanin extraction [48].

In this study, RSM was used to study the effect of salinity, glucose concentration (mixotrophic and heterotrophic conditions), and light color, as well as the combinatorial effects of these input parameters on *Spirulina* biomass productivity (quantity) and biomass composition (quality). For this purpose, both an in-house illumination system for the realization of different light colors with constant photon flux density, and a screening cultivation system based on small-scale cell culture flasks, were realized. The statistical analysis, based on 144 cultivations performed under varying process conditions, yielded a comprehensive knowledge on interaction effects of the process parameters (variables) on the process responses of biomass productivity and the product quality of *Spirulina* biomass.

## 2. Materials and Methods

The experiments were conducted in the experimental facilities of the Institute of Natural Materials Technology (TU Dresden, Dresden, Germany) for a period of 8 months during 2020–2021. The following methods were used to investigate, in detail, the influence of the process parameters light color, salinity, and glucose concentration (mixotrophic growth) on the biomass productivity, cellular protein, and phycocyanin content of *Spirulina*. The approach is intended to prove the hypothesis that a well-balanced combination of the above-mentioned process parameters has a positive effect on the biomass quantity and quality.

### 2.1. Microorganism

*A. platensis* PCC7345 was obtained from the Pasteur Culture Collection (PCC, Paris, France). Stock cultures were grown in a sterile filtrated modified Zarrouk medium containing (g/L): 16.8 NaHCO_3_ (99.5%, VWR Chemicals, Darmstadt, Germany), 0.5 K_2_HPO_4_ (99%, Carl Roth, Karlsruhe, Germany), 2.5 NaNO_3_ (99.9%, VWR chemicals, Darmstadt, Germany), 1.0 K_2_SO_4_ (99.5%, VWR chemicals, Darmstadt, Germany), 1.0 NaCl (99%, Carl Roth, Karlsruhe, Germany), 0.2 MgSO_4_∙7H_2_O (99%, Carl Roth, Karlsruhe, Germany), 0.04 CaCl_2_∙2H_2_O (99%, Carl Roth, Karlsruhe, Germany) [11], and 100 µL/L of Hutner’s trace element solution [49]. *A. platensis* stock cultures were maintained in 300 mL Erlenmeyer flasks at 26 °C, 150 rpm, and 75 µmol/m^2^s fluorescent light (light/dark cycles of 16/8 h, WB750, Mytron Bio- und Solartechnik GmbH, Heilbad Heiligenstadt, Germany). Liquid stock cultures were sub-cultivated every two weeks to prevent aging and cell death.

### 2.2. Light Panels

**Figure 1:**
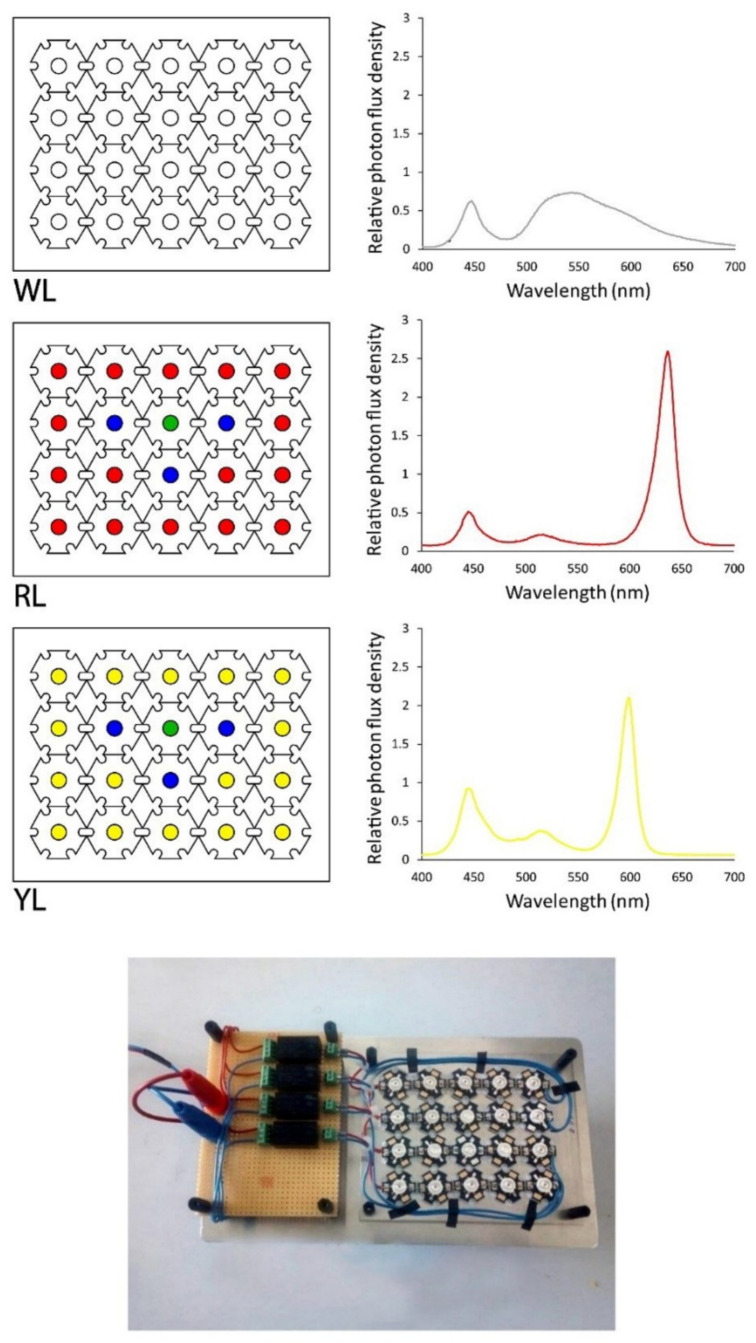
Arrangement of LEDs on light panels with customized spectral composition (photograph below graphs). The LED panels were named WL (white light panel), RL (red light panel), and YL (yellow light panel).

To customize the spectral lighting conditions, single high-power LEDs (3 W, 2.3–3.5 V, 700 mA, opening angle of 120°, World Trading Net GmbH & Co. KG, Bleicherode, Germany) were purchased in white (5000–7000 °K), blue (470 nm), yellow (590 nm), red (625 nm), and green (525 nm). Twenty LEDs using the ratio recommended by Mao and Guo [37] were arranged on three light panels (L) in the following configuration: WL) 100% white; RL) 80% red, 15% blue, 5% green; and YL) 80% yellow, 15% blue, 5% green. The spectral composition of each light panel was measured with a USB-650 spectrometer (Ocean Optics, Orlando, FL, USA), and was normalized to improve the comparison as visualized in Figure 1.

### 2.3. Adaptation and Inoculation of Cultivation Units

**Figure 2:**
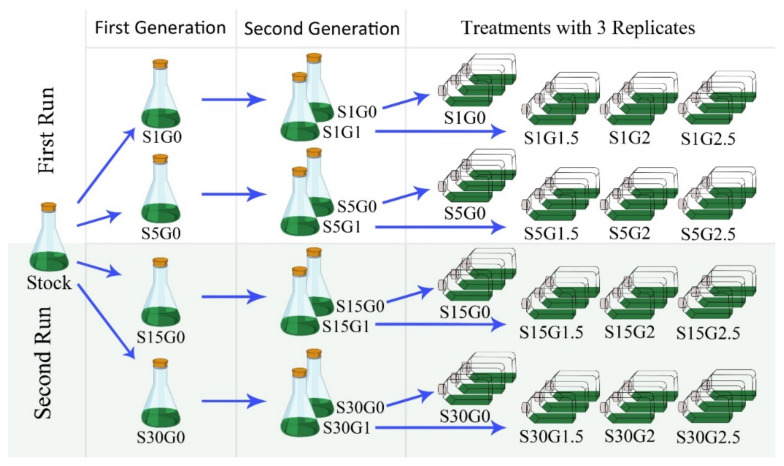
Routine for the adaptation of *Arthrospira platensis* (*Spirulina*) with varying concentrations of sodium chloride (S) and glucose (G) before each experimental run.

A gradual preadaptation of *Spirulina* using varying concentrations of sodium chloride (S) and glucose (G) was performed before each experimental run under continuous light (white fluorescent light, 100 µmol/m^2^s, 30 °C, WB750, Mytron Bio- und Solartechnik GmbH, Heilbad Heiligenstadt, Germany) and axenic conditions (Figure 2). First, pre-cultures were adapted to 1, 5, 15, 30 g/L of S (3–5 days depending to the amount of S), whereas the second pre-cultures were adapted to 0 or 1 g/L of G (3 days). To adjust the desired concentrations of S and G for the experimental runs, stock solutions of NaCl 200 % (99%, Carl Roth, Karlsruhe, Germany) and glucose 100 % (99.5%, Carl Roth, Karlsruhe, Germany) were added to the modified Zarrouk medium described in Section 2.1. The initial pH was adjusted to 9.5 by adding 1 N sodium hydroxide solution (99.8%, Carl Roth, Karlsruhe, Germany). Secondary pre-cultures growing at the log phase were used to inoculate the experimental runs in cell culture bottles at 0.1 g/L (200cc filter screw cap cell culture bottles, Greiner Bio-One, Frickenhausen, Germany). The final working volume of each bottle was adjusted to 50 mL. The dimensions of the bottles were 8 × 3.5 × 13 cm, yielding an illuminated surface of 45.5 cm^2^. This costume-made screening setup allowed performing of 24 parallel cultivations at homogenous light color and light intensity conditions, and formed the basis for the statistical analysis of biomass productivity and quality.

### 2.4. Experimental Conditions at Screening Scale for the Design of Experiments Approach

**Figure 3:**
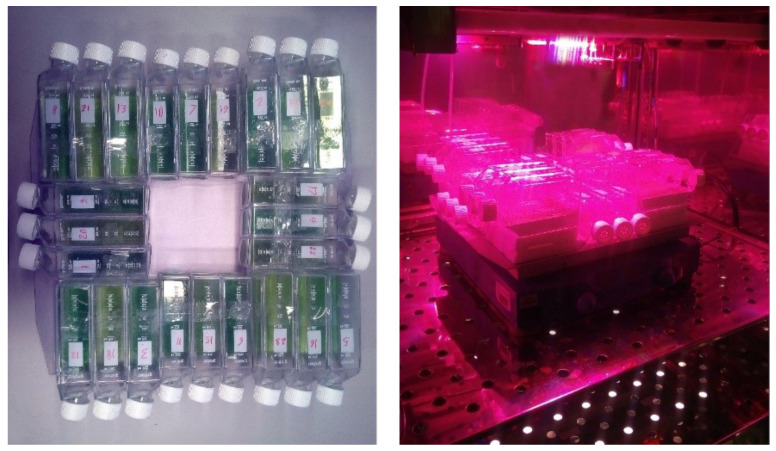
Arrangement of the experimental unit for the design of experiments approach in the incubator. The photograph shows the exposure of 24 culture bottles to the RL.

A total of 144 assays were performed with six runs (2 runs for each light panel with 24 assays). The cell culture bottles were fixed on a rotary platform shaker with a speed of 200 rpm in a controlled temperature environment at 30 °C [40]. In order to provide an equal photon flux density of 150 µmol/m^2^s for all bottles, the distance of the light source was adapted to ensure that the experimental runs were under comparable light quantity conditions [50] (Figure 3). The photon flux density of the light panels was measured using a PAR-quantum sensor DK-PHAR 2.010BS (Deka Sensor + Technologie Entwicklungs- und Vertriebgesellschaft mbH, Teltow, Germany). The light panels were exposed to light/dark cycles of 16/8 h.

### 2.5. Sampling and Analytical Methods

Each experimental screening run was performed for 5 days. Sampling was conducted daily; however, the analysis of biochemical composition (quality) was performed at the end of the dark cycle on the third cultivation day. This time was determined in pre-tests (data not shown) to ensure there was non-limited growth at the end of the exponential growth phase for all tested conditions.

### 2.6. Determination of Dry Weight Concentration

The biomass dry weight concentration was measured as changes in optical density (OD_750_) using a spectrophotometer (Genesys 150, ThermoFisher Scientific, Waltham, MA, USA). Routines for cell dry weight determination and optical density correlation were recently described by Franke et al. [51].

### 2.7. In-Vivo Phycocyanin Quantification of Suspended Cells

The quantification of the cellular phycocyanin content, c_PC_ (mg/g), of *Spirulina* was performed as recently described by Franke et al. [51]. In brief, culture samples were diluted using saline solution (0.9 % NaCL) to a final OD_750_ of 0.1 in a 3 mL cuvette that was clear on all sides, and made from polystyrene. The intracellular phycocyanin was excited with light in the spectral range of 600–630 nm, and the maximum of phycocyanin absorption (step with 5 nm, measurement speed: 1200 nm/min), using a fluorescence spectrometer (LS-55, PerkinElmer Inc., Waltham, MA, USA). Fluorescence emission was detected in the range of 650–670 nm with a peak intensity (I_f, max_) at 655 nm. I_f, max_ was detected in triplicate, and used to calculate the cellular phycocyanin content following Equation (1).

_CPC_ = 0.4484 × I_f max_ (1)

### 2.8. Quantification of Cellular Protein Content and pH Measurement

At the end of the dark cycle on the third cultivation day, 10 mL of cell suspensions were taken from each cell culture bottle to determine the protein content of the biomass. First, the pH of the medium was measured using a pH meter (Xylem Analytics Germany Sales GmbH & Co. KG, Weilheim, Germany), followed by biomass separation at 10,000 rpm (3–30 ks, Refrigerated Centrifuge, Sigma Laborzentrifugen GmbH, Osterode, Germany) for 20 min at 4 °C. The pelleted biomass was washed with 1 mL saline water (same salinity as respective culture medium). This washing step was repeated a total of three times [52]. The collected biomass was freeze-dried (LSCplus, Martin Christ Gefriertrocknungsanlagen GmbH, Osterode, Germany) to analyze the protein content with a Lowry assay using the procedure described by Slocombe et al. [53].

### 2.9. Design of Experiment—Modulation of Responses

The design of experiments approach was planned as a full factorial design of 3 × 4 × 4 (Table 1). All experiments were carried out in triplicate, yielding a total of 144 experimental runs to identify the impact of the three process variables: (1) light quality (L), (2) sodium chloride concentration (S), and (3) glucose concentration (G), on the respond parameters of the biomass dry weight concentration (g/L), cellular phycocyanin content (mg/g), and protein content (%) in batch cultures. Each experimental run was assigned an ID, which is structured as follows: YLS30G0 indicates yellow light conditions (YL), a salinity (S) of 30 g/L, and a glucose concentration (G) of 0 g/L.

The statistical effect of each factor was determined with the analysis of variance (ANOVA) method using Design-Expert (Version 12) at a confidence coefficient level of α = 0.05. The model fit accuracy was assessed based on the model validity (lack of fit) and the explained variation (R^2^). To validate the quality of the DOE models, experiments were conducted under the predicted optimal process conditions, and compared to the predicted model outcomes.

## 3. Results

The experimental basis for the RSM approach was provided by the 144 cultivations carried out under variation of the input variables: light color, salinity, and glucose concentration. In the following sections, the results of process analytics, as well as the modeling and the validation of the model prediction, are presented.

### 3.1. DOE—Output Responses and Model Fitting

The analyzed mean output parameter presented in Table 2 shows a huge variance in the obtained biomass dry weight concentration, as well as the cellular protein and phycocyanin content, dependent on the applied process conditions. The biomass dry weight concentration after three days of cultivation ranged from 0.65 g/L (YLS30G0) to 1.25 g/L (RL15S2.5G); the cellular phycocyanin content from 48 mg/g (RL5S2.5G) to 114 mg/g (YL1SG0); and the protein content from 23% (RL30S2.5G and WL30S2.5G) to 64% of freeze-dried biomass (YL1S0G).

The experimental data were used to identify model equations for each light color condition. The multiple regression analyses of variance for the significance of the different-order polynomial equations of experimental data are shown in Table 3. For the lack-of-fit test, the *p* > 0.05 indicates that the model is significant at a 95% confidence interval. The sequential sum of squares for the two-factor interaction (2FI) was fitted to the target parameter “phycocyanin content,” whereas the cubic model was fitted for the target parameter “biomass concentrations” and “protein content,” yielding an estimated R^2^ of 0.66, 0.85, and 0.77, respectively.

### 3.2. Model Development and RSM

The results of ANOVA tests for the effect of DOE parameters and their interactions are presented in Table 4, and indicate an interaction effect between the studied input parameters. In the cubic model accounting for the biomass concentration of S, G, L, SG, and SL: S^2^, S^2^G, S^2^L, and S^3^ were identified as significant model terms (*p* < 0.05); in the 2FI model applied for phycocyanin S, G, L, SL, and GL, and in the cubic model used to describe the protein content of S, L, SL, GL, and SGL: S^2^L and S^3^ were identified as significant model terms (*p* < 0.05).

The optimized and reduced models for each light color condition produced by removing insignificant interactions are shown in Equations (2)–(10), where S and G in the equations represent the values of sodium chloride (g/L) and glucose (g/L), respectively.

Red light conditions:

Biomass (g/L) = 0.774378 + 0.007S + 0.153028G + 0.003046SG + 0.001222S^2^ − 0.046097G^2^ − 0.000302S^2^G + 0.001913SG^2^ − 0.00005S^3^ (2)

Phycocyanin (mg/g) = 58.78636 + 0.356963S− 2.34278G(3)

Protein (%) = 41.43973 + 1.64206S + 1.22094G − 0.089108SG − 0.217611S^2^ − 0.272088G^2^ + 0.004940S^3^ (4)

White light conditions:

Biomass (g/L) = 1.00961 − 0.03227S + 0.153028G + 0.003046SG + 0.002291S^2^ − 0.0461G^2^ − 0.0003S^2^G + 0.001913SG^2^ − 0.00005S^3^ (5)

Phycocyanin (mg/g) = 86.46885 − 0.5755S − 0.50084G(6)

28.39144 + 3.19007S + 5.81073G − 0.226657SG − 0.248256S^2^ − 0.705852G^2^ + 0.004940S^3^ (7)

White light conditions:

Biomass (g/L) = 0.78752 − 0.03441S + 0.153028G + 0.003046SG + 0.002506S^2^ − 0.0461G^2^ − 0.000302S^2^G + 0.001913SG^2^ − 0.00005S^3^ (8)

Phycocyanin (mg/g) = 114.2279 − 1.02457S − 6.47468G (9)

Protein (%) = 62.45694 + 0.303057S − 11.10308G + 0.095447SG − 0.192155S^2^ + 3.33310G^2^ + 0.004940S^3^ (10)

**Figure 4:**
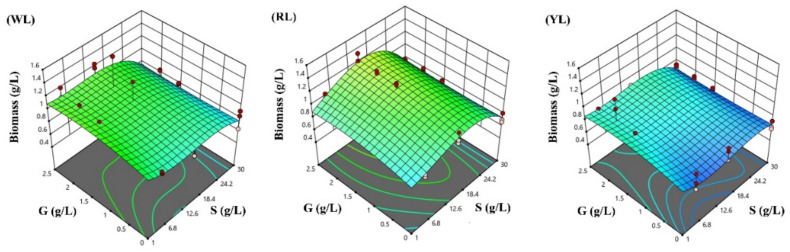
Response surface methodology presenting the effects of sodium chloride (S) and glucose (G) concentration on *Spirulina* biomass concentration after three days of cultivation (g/L): (**WL**) white light panel, (**RL**) red light panel, and (**YL**) yellow light panel; the circles are the designated points.

**Figure 5:**
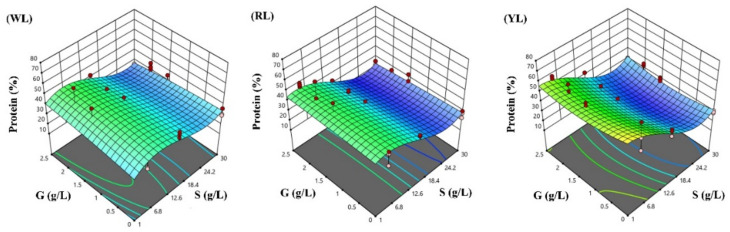
Response surface methodology presenting the effects of sodium chloride (S) and glucose (G) concentrations on the *Spirulina* protein content (%): (**WL**) white light panel, (**RL**) red light panel, and (**YL**) yellow light panel; the circles are the designated points.

**Figure 6:**
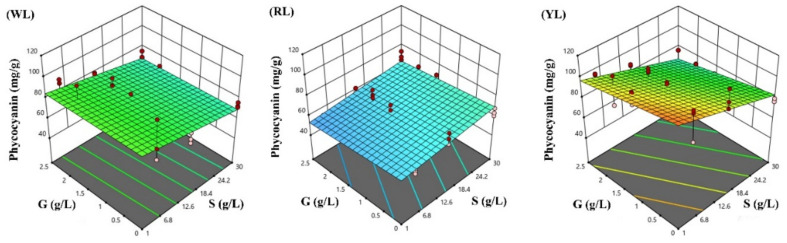
Response surface methodology presenting the effects of sodium chloride (S) and glucose (G) concentrations on the *Spirulina* phycocyanin content (mg/g): (**WL**) white light panel, (**RL**) red light panel, and (**YL**) yellow light panel; the circles are the designated points.

The response surfaces for the biomass concentration, and protein and phycocyanin content in the three lighting conditions studied were developed following the optimized polynomial models, and are visualized in Figure 4, Figure 5 and Figure 6.

The ability of microalgae to use different light spectra is related to the photosynthetic pigment composition [29], and the availability of photons due to light attenuation in suspension [54]. Under phototrophic conditions (0G), and with typical salinity (1S), the highest biomass concentration was obtained under WL (1.03 g/L) conditions, whereas the biomass concentration achieved with RL (0.85 g/L) and YL (0.66 g/L) was reduced in a similar range. Previous studies that have investigated the dependence of biomass productivity on light color have presented far different results. In general, light in the blue spectral range shows good penetration in water, and can be efficiently absorbed by chlorophyll and carotenoids, but a too high dose of blue light can result in non-photochemical quenching processes [55]. Light in the red spectral range can be efficiently absorbed by chlorophyll and phycocyanin [56,57], resulting in high biomass concentrations and growth rates, reported in recent studies [11,34,58,59,60].

Chainapong et al. [36] reported a higher growth rate of *Spirulina* under white light in comparison to red and yellow lights produced by plastic filters. Ravelonandro et al. [61] used colored polyethylene films to obtain different light spectra, and found that the final biomass of *A. platensis* exposed to green or white light was higher compared to red light. In contrast, Mao and Guo [37] reported a facilitated growth rate under red-dominated light treatments. However, they used a white light source with a higher blue spectral component and supplementary CO_2_ bubbling compared to this study. Tayebati et al. [60] reported a higher biomass concentration under red LED light conditions compared to white, yellow, and blue light. Park and Dinh [62] observed no significant difference in the growth of *A. maxima* cultivated under red (660 nm) and white light, whereas Zittelli et al. [63] found an improved biomass productivity of *Spirulina* under orange light (620 nm) compared to white and blue light conditions. The severely disparate results of these studies may be attributed to the different experimental setups, different species, and various sources and wavelengths of light. The high pH value under red light conditions (11.15) observed in this study indicates a high photosynthetic activity; however, a beneficial growth effect of red-dominated light could not be confirmed in this study, as the biomass concentration of *Spirulina* was reduced by approx. 17.5% under phototrophic conditions (0 g/L glucose), and 1 g/L NaCl under RL conditions, compared to WL.

According to the RSM prediction, the biomass concentration can be boosted by the addition of glucose under mixotrophic conditions with the following configuration of cultivation parameters for each lighting condition (after 3 days of cultivation): 1.21 g/L for RL panel adjusting a salinity of 16.85 g/L and 2.5 g/L glucose; 0.89 g/L for WL adjusting a salinity of 1 g/L and 2.5 g/L glucose; and finally, 0.89 g/L at YL adjusting 1 g/L salinity and 1.76 g/L glucose, respectively. However, the predicted protein content under RL (28.56%) is significantly lower than under WL (38.98%) and YL (53.52%), respectively.

In contrast, the lowest biomass concentration is achieved at the following parameter configuration: 0.67 g/L for YL at 0.89 g/L glucose and 4.96 g/L NaCl; 0.88 g/L for WL at 0 g/L glucose and 6.30 g/L NaCl; and 0.89 g/L at 0 g/L glucose and 30 g/L NaCl. The RSM coefficients of glucose remained equal for the different light panels used, indicating that the interaction between the light color and glucose concentration on biomass production of *Spirulina* was not significant, i.e., the color of the light did not have a significant effect on the mixotrophic growth of *Spirulina*.

In recent studies, the effect of increasing NaCl on *Spirulina* production has been assessed [19,64,65,66], indicating that the growth of *Spirulina* remains stable, using NaCl concentrations up to 13 g/L, whereas higher salinities resulted in a reduction in growth rates. In the literature, the growth reduction at high salinity conditions is mainly attributed to two effects. The first effect is an increase in maintenance costs: the best salinity level for phytoplankton is equal to the salinity of their cytoplasm; higher or lower values are controlled by osmotic mechanisms. In the case of high salinity, the first strategy is the extrusion of sodium ions by using energy; alongside this, a high amount of compatible osmolytes accumulate inside of the cell to balance the osmolality [67]. Additionally, *Spirulina* produces a huge amount of extracellular polymeric substances, which enhances salt tolerance [54,60]. In this manner, along with the energy used for osmotic adjustments, the cell protects sub-cellular structures from damage. The second effect is a reduced photosynthetic activity, photoinhibition, and an increase in respiration, which were reported in recent studies [24,50,68,69]: salt stress led to a 40% loss of a thylakoid membrane protein known as D1, whereas salinity stress proportional to the intensity of PAR blocked electron transport, and inhibited PSII electron transport [69].

In contrast, Dhiab et al. [70] reported that an elevated salt concentration, even at 500 mM (29.22 g/L), enhanced the growth and photosynthetic efficiency of *Spirulina*. This discrepancy in the results may be explained by the fact that the authors used a light intensity of 20 µmol/m^2^s, which is much lower than that in the above studies [25,69,71]. Low light intensity seems to be a suitable approach of maintaining the vitality and growth of the cells under raised salinity; however, high light intensities result in reduced growth due to photoinhibition.

In this study, the average biomass under RL with 1 g/L and 5 g/L NaCl (assays 49–60 and 61–72) was 25% and 11% lower compared to the same conditions in WL (assays 1–12 and 13–24). In contrast, the average biomass under RL with 15 g/L NaCl (assays 73–84) was 12% higher than WL (assays 25–36). The optimum pH for *Spirulina* growth is 10.5. At a pH close to 11, cells undergo deterioration [72], a possible reason for the poorer performance of the RL with 1 and 5 g/L NaCl (average pH of 10.93) than WL with 1 and 5 g/L NaCl (average pH of 10.64) and RL with 15 g/L (average pH of 10.38).

Mixotrophic conditions using glucose as an additional carbon source enhanced the biomass productivity under varying light color conditions. An inhibitory effect was not observed under any of the analyzed conditions using glucose at a concentration up to 2.5 g/L. Results of the current study are consistent with earlier research that studied the growth of *Spirulina* cultured with different organic carbon sources [34,36,73,74,75]. Rasouli et al. [75] reported that the biomass productivity of *Spirulina* was significantly reduced by the addition of more than 4 g/L glucose. However, *Spirulina* can survive in media containing up to 20 g/L of glucose. Therefore, these authors recommended applying glucose in the concentration range of 0–1.5 g/L. Bachchhav et al. [34] observed that a mixotrophic culture using yellow LED light achieved a higher final biomass concentration compared to red and white LEDs. This difference could be attributed to the fact that their study lasted 10 days with a final biomass of 6.6 g/L, with a poor level of details.

In mixotrophic culture conditions, the adverse effect of high salinity up to 15 g/L is mediated by heterotrophic growth. Rasouli et al. [75] reported that the osmolarity of *Spirulina* was significantly improved by the addition of glucose. Moreover, the results of current study are in line with Mata et al. [27], who reported an improved biomass productivity of *Spirulina* in mixotrophic conditions (1 g/L glucose) with a reduced NaCl content.

*Spirulina* cultivation can be an alternative method to produce proteins for food or feed industries; thus, the protein content is an important factor when considering the nutritional value of *Spirulina* [76]. At phototrophic conditions with a normal NaCl content (1 g/L), the highest protein content was obtained under YL (64%), and the protein content under RL (44%) was higher than under WL (31%). The higher protein content under YL probably is the result of the higher phycocyanin content and stable pH conditions achieved at this parameter combination. Furthermore, our results also confirm the hypothesis that the protein level in Spirulina is favored by slow growth [77]. Only few studies have been carried out addressing the effect of light color on the protein content of *Spirulina*. For instance, Ravelonandro et al. [61] observed a higher protein content under white compared to green, red, and blue light, respectively. In Milia et al. [78], the protein content of *Spirulina* was higher under blue and white fluorescent light compared to orange light in the studies by Markou [11] and da Fontoura Prates et al. [10], and the protein productivity under red or red and green LEDs was higher than under white LEDs. However, the spectral conditions of the light sources were not presented by the authors, which makes it difficult to interpret and compare the results.

According to RSM prediction, the highest cellular protein levels will be achieved under YL (62.57%), and then in WL (46.43%), both under 6.57 g/L NaCl and 2.50 g/L glucose. Under RL, the highest protein level (45.53%) will be achieved under 3.99 g/L NaCl and 1.59 g/L glucose. The lowest protein levels will be achieved under RL (19.70%) with 26.19 g/L NaCl and 2.40 g/L glucose, then in YL (21.41%) under 24.76 g/L NaCl and 1.31 g/L glucose, and in WL (25.59%) under 26.92 g/L NaCl and 2.50 g/L glucose.

The results of this study are in agreement with other studies [64,65,79,80]. Under stress, microalgae undergo significant metabolic and physiological changes, yielding an increase of cellular lipids, carbohydrates, carotenoids, and antioxidant enzymes [81]. Crucial alterations occur in the *Spirulina* proteome under salinity stress, yielding a reduction in the cellular protein content [82]. Previous studies have demonstrated the complete blockage of protein synthesis alongside carbohydrate incensement in cyanobacteria after salt stress [83,84]. According to Vonshak [54], in the presence of 0.5 mol/L (29.3 g/L) NaCl in the medium, the carbohydrate content of *Spirulina* biomass reached up to 64.4%. Similarly, Mary Leema et al. [85] reported a 79 % increase in the carbohydrate content of the biomass when *Spirulina* was cultivated in pre-treated seawater. Accordingly, the protein reduction observed in our study might be related to both an increase in carbohydrate and lipid production, and the suppression of protein synthesis.

In phototrophic conditions G (0 g/L) and normal S (1 g/L), the highest phycocyanin content was achieved under YL (114 mg/g), and the phycocyanin content under WL (87 mg/g) was higher than RL (63 mg/g). According to the model prediction, the highest phycocyanin content will be achieved under YL (113.20 mg/g), followed by WL (85.90 mg/g), both under 1 g/L NaCl without glucose. Under RL, the highest phycocyanin (69.5 mg/g) will be achieved under 30 g/L NaCl without glucose. The lowest phycocyanin contents will be achieved under RL (53.29 mg/g) exposed to 1 g/L NaCl and 2.5 g/L glucose, then in YL (67.30 mg/g), and WL (67.95 mg/g), both under 30 g/L NaCl and 2.5 g/L glucose.

Phycobilisomes, the main light-harvesting complexes in cyanobacteria, are strongly influenced by environmental changes [86]. Light is the main environmental parameter that affects the overall growth rate for photoautotrophic microorganisms [87]. The two main pigments of *Spirulina* are chlorophyll a, with an absorption peak of 429 and 662 nm [88], and phycocyanin, with an absorption peak of 620 nm [57], depending on the relative light emitted from each panel (Figure 1). The light emitted by the RL panel shows a better overlap with chlorophyll a absorption, whereas the YL panel shows a better overlap with the phycocyanin absorption range. Therefore, this spectral fitting of the light source and photosynthetic pigment absorption might be responsible for the increased phycocyanin content using YL. These results are in accordance with Bachchhav et al. [34], who investigated the phycocyanin content of *Spirulina* under different LED colors, and found an increase in the phycocyanin content in cells exposed to yellow LED light. Tayebati et al. [60] found the highest phycocyanin content under monochromatic red LED light (with 660 nm peak), which was higher than white, yellow (with 590 nm peak), and blue light conditions. Milia et al. [78], investigated the effect of white, orange, and blue light treatment on the phycocyanin content of *A. platensis* M, *A. platensis* M2M, and *A. maxima*, indicating that various *Spirulina* strains show different responses in the cellular phycocyanin content towards changing light color conditions.

In this study, we found that mixotrophic cultivation had a significant negative effect on the phycocyanin content, but the effect of high salinity was even higher. Phycocyanin is an accessory pigment, and photosynthesis is essential for phycocyanin biosynthesis [39]. Glucose is an effective substrate for respiration that can inhibit the photosynthetic processes [89]. Consequently, during mixotrophic cultivation, the metabolism may switch between phototrophic or heterotrophic [90]. Chen and Zhang [90] reported a constant phycocyanin content during phototrophic cultivation. In contrast, with a mixotrophic culture, the phycocyanin content was affected by the glucose concentration and cell intensity, in which mutual shading and photolimitation occurs in dense cultures. Indeed, at high glucose concentrations (more than 1 g/L) or high cell densities, *Spirulina* tends towards a heterotrophic metabolism, and the phycocyanin content decreases rapidly [90,91]. In contrast, Bachchhav et al. [34] reported a higher phycocyanin content under mixotrophic conditions using a low initial glucose concentration of 1 g/L, which could lead to the metabolization of glucose at an early stage in the experiment. Chainapong et al. [36] detected a similar phycocyanin content in mixotrophic conditions under yellow and red light, probably due to the use of filtered sunlight resulting in spectral overlaps in light colors.

When the amount of NaCl was increased, the mean cellular phycocyanin concentration decreased under WL (−10% in 15 g/L NaCl to −20% in 30 g/L NaCl) and YL (−10% in 15 g/L NaCl to −30% g/L NaCl), respectively, but it increased under RL (+8.6% in 15 g/L NaCl to +10% in 30 g/L NaCl). The reduction in the phycocyanin content at high salinities has been confirmed in many previous studies [19,80,92]. According to Rafiqul et al. [64], the phycocyanin content of *Spirulina* decreased, whereas the carotenoid and lipid content increased under salt stress conditions. Lu and Vonshak [93] reported that the amount of chlorophyll remained stable, whereas the amount of phycocyanin decreased to 50% of control treatment when *Spirulina* was exposed to 46.75 g/L sodium chloride for 12 h.

### 3.3. Optimization and RSM Validation

In order to validate the predictive power of the RSM models, experiments were performed using the predicted optimum conditions of glucose concentration and salinity for each of the spectral lighting conditions.

The predicted and measured parameters under optimized variable conditions are shown in Table 5. The goal of this study was to find the best-balanced growth conditions for both a high quantitative biomass productivity and a high-quality biomass composition, represented by a high cellular protein and phycocyanin content. The optimized process parameter configurations which were suggested by the RSM model are YL using 1.00 g/L NaCl and 0.88 g/L glucose, WL using 5.30 g/L NaCl and 2.46 g/L glucose, and RL at 9.10 g/L NaCl and 1.30 g/L glucose.

Biomass concentration and protein content were close to the predicted values; however, the cellular phycocyanin contents under WL and RL were higher than the predicted response. As observed in this study, the phycocyanin content is a highly dynamic parameter compared to the biomass concentration and protein content. For a more accurate prediction of phycocyanin content, more variables would need to be added to the RSM model, such as the initial phycocyanin concentration and the culture medium pH.

## 4. Conclusions

In this study, the authors intended to optimize *Spirulina* batch cultivation by balancing the process parameters of light color, salinity, and glucose concentration in order to produce a high quantity of biomass (productivity) with a high quality (maximum protein and phycocyanin contents). By developing a screening system that allows 24 parallel cultivations under homogeneous light conditions, and a fine-tuned analysis for small sample volumes (especially protein and phycocyanin content), it was possible, for the first time, to identify the impact of single factors, as well as combinatorial effects, on product quantity and quality. RSM proved to be a powerful tool for model prediction under varying spectral light conditions (RL, WL, YL), modes of trophic growth (phototrophic vs. mixotrophic), and salinities. The RSM models obtained are useful for developing *Spirulina* production in photobioreactors with artificial light, and optimizing the growing conditions for the phototrophic or mixotrophic cultivation of *Spirulina* with brackish and saline water supplies.

All the experimental variables in the cultivation of *Spirulina* had an effect on the biomass concentration and phycocyanin content, but glucose did not have a significant effect on the protein content. WL led to the highest biomass concentration after three days of cultivation, but higher protein and phycocyanin contents were achieved under YL light. A mixotrophic culture increased the biomass concentration, whereas increasing salinity decreased the biomass, phycocyanin, and protein contents. The main hypothesis of this study was that *Spirulina* production could be increased by combining LEDs with yellow light predominance under optimal mixotrophic and salinity conditions. Although the highest level of biomass production was observed in predominantly red light, quality indicators were higher in algae produced with yellow light. Consequently, in line with the optimized conditions, we recommend using YL1.00S0.88G, WL5.30S2.46G, and RL9.10S1.30G, respectively, to produce the highest level of biomass with the highest quality.

For further studies, it is recommended to use the spectral composition of light panels instead of the number ratio of LEDs. In addition, performing experiments in a pH-controlled system will have more accurate results, and larger scales in semi-batch or continuous cultures with commercial mediums are needed to bridge the gap between laboratory results to industrial use.

## Figures and Tables

**Table 1:** Experimental independent variables and levels.

Factors	Levels			
**Light panel (L)**	White (W)	Red (R)	Yellow (Y)	-
**Sodium chloride concentration (S, g/L)**	0	5	15	30
**Glucose concentration (G, g/L)**	0	1.5	2	2.5

**Table 2:** The response of *Arthrospira platensis (Spirulina)* to different levels of sodium chloride (S) and glucose (G) concentration under different light panels (L) where the dominant color was white (W), red (R), and yellow (Y); standard deviation is calculated from three independent experimental runs (*n* = 3).

Assays	Treatment			Responses			
	L	S (g/L)	G (g/L)	Biomass (g/L)	Phycocyanin (mg/g)	Protein (%)	pH
1–3	W	1	0	1.03 ± 0.01	87 ± 7	31 ± 7	10.85 ± 0.06
4–6	W	1	1.5	1.16 ± 0.05	82 ± 8	33 ± 2	10.74 ± 0.12
7–9	W	1	2	1.11 ± 0.11	85 ± 2	33 ± 4	10.69 ± 0.19
10–12	W	1	2.5	1.10 ± 0.10	88 ± 4	44 ± 5	10.54 ± 0.11
13–15	W	5	0	0.93 ± 0.02	89 ± 19	33 ± 1	10.68 ± 0.16
16–18	W	5	1.5	1.02 ± 0.04	81 ± 3	48 ± 8	10.58 ± 0.05
19–21	W	5	2	1.07 ± 0.05	83 ± 11	49 ± 9	10.53 ± 0.14
22–24	W	5	2.5	1.07 ± 0.13	82 ± 12	42 ± 1	10.48 ± 0.21
25–27	W	15	0	0.77 ± 0.01	72 ± 5	42 ± 3	10.38 ± 0.02
28–30	W	15	1.5	1.03 ± 0.29	76 ± 4	37 ± 5	10.16 ± 0.06
31–33	W	15	2	1.15 ± 0.33	80 ± 4	37 ± 2	10.18 ± 0.03
34–36	W	15	2.5	1.12 ± 0.16	77 ± 3	39 ± 3	10.18 ± 0.02
37–39	W	30	0	0.79 ± 0.15	73 ± 2	32 ± 4	9.99 ± 0.04
40–42	W	30	1.5	0.73 ± 0.11	64 ± 2	30 ± 5	9.80 ± 0.02
43–45	W	30	2	0.80 ± 0.07	67 ± 3	34 ± 3	9.61 ± 0.37
46–48	W	30	2.5	0.69 ± 0.03	71 ± 4	23 ± 2	9.8 ± 0.03
49–51	R	1	0	0.85 ± 0.03	63 ± 10	44 ± 5	11.15 ± 0.08
52–54	R	1	1.5	0.94 ± 0.05	57 ± 4	42 ± 5	10.96 ± 0.05
55–57	R	1	2	0.86 ± 0.03	57 ± 6	41 ± 2	10.82 ± 0.08
58–60	R	1	2.5	0.87 ± 0.03	56 ± 2	42 ± 4	10.76 ± 0.07
61–63	R	5	0	0.80 ± 0.02	58 ± 2	40 ± 8	11.13 ± 0.14
64–66	R	5	1.5	0.93 ± 0.03	50 ± 4	44 ± 8	10.94 ± 0.04
67–69	R	5	2	0.98 ± 0.01	49 ± 3	48 ± 14	10.8 ± 0.02
70–72	R	5	2.5	0.98 ± 0.05	48 ± 5	48 ± 2	10.9 ± 0.05
73–75	R	15	0	0.97 ± 0.09	69 ± 6	38 ± 1	10.33 ± 0.17
76–78	R	15	1.5	1.22 ± 0.02	62 ± 4	32 ± 3	10.48 ± 0.04
79–81	R	15	2	1.16 ± 0.10	66 ± 4	34 ± 1	10.35 ± 0.16
82–84	R	15	2.5	1.25 ± 0.08	57 ± 5	28 ± 4	10.38 ± 0.07
85–87	R	30	0	0.73 ± 0.07	64 ± 4	27 ± 4	9.94 ± 0.01
88–90	R	30	1.5	0.71 ± 0.06	66 ± 3	27 ± 6	9.80 ± 0.02
91–93	R	30	2	0.75 ± 0.04	63 ± 4	25 ± 1	9.81 ± 0.01
94–96	R	30	2.5	0.74 ± 0.03	69 ± 5	23 ± 2	9.83 ± 0.04
97–99	Y	1	0	0.66 ± 0.13	114 ± 5	64 ± 10	10.18 ± 0.14
100–102	Y	1	1.5	0.85 ± 0.16	106 ± 5	49 ± 10	10.27 ± 0.21
103–105	Y	1	2	1.03 ± 0.14	96 ± 10	52 ± 6	10.43 ± 0.20
106–108	Y	1	2.5	0.85 ± 0.11	101 ± 7	58 ± 5	10.22 ± 0.12
109–111	Y	5	0	0.82 ± 0.13	94 ± 16	55 ± 14	10.32 ± 0.24
112–114	Y	5	1.5	0.82 ± 0.06	96 ± 7	52 ± 3	10.14 ± 0.03
115–117	Y	5	2	1.00 ± 0.21	90 ± 14	54 ± 7	10.43 ± 4.95
118–120	Y	5	2.5	0.82 ± 0.03	94 ± 3	55 ± 3	10.14 ± 0.03
121–123	Y	15	0	0.78 ± 0.10	108 ± 9	42 ± 4	10.34 ± 0.03
124–126	Y	15	1.5	0.74 ± 0.04	90 ± 1	34 ± 5	10.16 ± 0.05
127–129	Y	15	2	0.75 ± 0.09	92 ± 2	32 ± 9	10.17 ± 0.07
130–132	Y	15	2.5	0.87 ± 0.02	84 ± 5	36 ± 5	10.24 ± 0.06
133–135	Y	30	0	0.65 ± 0.07	79 ± 2	30 ± 1	10.11 ± 0.04
136–138	Y	30	1.5	0.66 ± 0.05	70 ± 2	29 ± 2	9.97 ± 0.02
139–141	Y	30	2	0.66 ± 0.06	71 ± 1	34 ± 1	9.96 ± 0.04
142–144	Y	30	2.5	0.71 ± 0.03	68 ± 7	28 ± 4	9.92 ± 0.04

**Table 3:** Analysis of variance of model statistics.

Source	SS	DF	MS	F-Value	*p*-Value	Adjusted R^2^
Biomass						
**Linear**	1.78	43	0.0413	3.82	<0.0001	0.42
**2FI**	1.59	38	0.0418	3.86	<0.0001	0.44
**Quadratic**	1.06	36	0.0294	2.72	<0.0001	0.55
Cubic *	0.4104	26	0.0158	1.46	0.0961	0.66
**Pure error**	1.04	96	0.0108			
**Phycocyanin**						
**Linear**	9166.21	43	213.17	4.94	<0.0001	0.68
2FI *	2033.28	38	53.51	1.24	0.1999	0.85
**Quadratic**	1862.87	36	51.75	1.2	0.2401	0.85
**Cubic**	1248.73	26	48.03	1.11	0.342	0.85
**Pure error**	4141.74	96	43.14			
**Protein**						
**Linear**	5052.45	43	117.5	3.74	<0.0001	0.52
**2FI**	3249.51	38	85.51	2.73	<0.0001	0.61
**Quadratic**	3204.69	36	89.02	2.84	<0.0001	0.61
Cubic *	1008	26	38.77	1.24	0.2277	0.73
**Pure error**	3012.54	96	31.38			

* Suggested model. SS sum of squares, DF degree of freedom, MS mean squares.

**Table 4:** The *p*-values and *F*-values of the parameters to biomass, phycocyanin, and protein response.

Source	Biomass Cubic Model		Phycocyanin 2FI Model		Protein Cubic Model	
	F-Value	*p*-Value	F-Value	*p*-Value	F-Value	*p*-Value
**Model**	14.23	<0.0001 *	87.72	<0.0001 *	19.02	<0.0001 *
**S-Sodium chloride**	79.71	<0.0001 *	70.42	<0.0001 *	219.56	<0.0001 *
**G-Glucose**	28.80	<0.0001 *	26.38	<0.0001 *	0.6894	0.408
**L-Light panel**	37.65	<0.0001 *	268.96	<0.0001 *	28.25	<0.0001 *
**SG**	4.06	0.0462 *	1.94	0.1662	2.71	0.1025
**SL**	5.79	0.0040 *	67.9	<0.0001 *	22.38	<0.0001 *
**GL**	0.0310	0.9695	8.53	0.0003 *	3.62	0.0296 *
**S^2^**	42.14	<0.0001 *			0.272	0.603
**G^2^**	2.41	0.1231			1.09	0.299
**SGL**	0.5577	0.5740			4.37	0.0147 *
**S^2^G**	8.53	0.0042 *			1.12	0.2914
**S^2^L**	16.89	<0.0001 *			10.16	<0.0001 *
**SG^2^**	2.35	0.1277			4.58	0.0344 *
**G^2^L**	0.1207	0.8864			2.89	0.0592
**S^3^**	7.18	0.0084 *			25.56	<0.0001 *
**G^3^**	1.49	0.2243			0.5553	0.4576

* represents *p* < 0.05.

**Table 5:** Predicted and experimentally determined number of responses under optimized variable conditions.

Optimum Conditions	Desirability	Responses					
		Biomass (g/L)		Phycocyanin (mg/g)		Protein (%)	
		Predicted	Actual	Predicted	Actual	Predicted	Actual
WL5.30S2.46G	0.50	1.07	1.09	82.18	108.74	46.13	45.64
RL9.10S1.30G	0.34	1.05	1.03	58.98	79.50	42.15	39.99
YL1.00S0.88G	0.55	0.86	0.91	106.96	115.68	55.06	51.09

## Data Availability

Raw data of this research are available by contacting the authors.
